# Thrombocytopenia Complicating Transcatheter Aortic Valve Implantation: Differences Between Two New-Generation Devices

**DOI:** 10.1007/s12265-021-10117-9

**Published:** 2021-03-15

**Authors:** Nicola Corcione, Simona Romano, Alberto Morello, Paolo Ferraro, Michele Cimmino, Michele Albanese, Martina Tufano, Daniela Capasso, Salvatore Buonpane, Salvatore Giordano, Martino Pepe, Giuseppe Biondi-Zoccai, Maria Fiammetta Romano, Arturo Giordano

**Affiliations:** 1 Unità Operativa di Interventistica Cardiovascolare, Pineta Grande Hospital, Castel Volturno, Italy; 2 Dipartimento di Medicina Molecolare e Biotecnologie Mediche, Università di Napoli Federico II, Pansini, 5, 80131 Napoli, Italy; 3 Unità Operativa di Emodinamica, Santa Lucia Hospital, San Giuseppe Vesuviano, Italy; 4 Dipartimento Cuore U.O.C. Cardiologia-UTIC, Pineta Grande Hospital, Castel Volturno, Italy; 5 Unità di Medicina di Laboratorio, Pineta Grande Hospital, Castel Volturno, Italy; 6 Division of Cardiology, Department of Emergency and Organ Transplantation, University of Bari, Bari, Italy; 7 Department of Medical-Surgical Sciences and Biotechnologies, Sapienza University of Rome, Latina, Italy; 8 Mediterranea Cardiocentro, Napoli, Italy

**Keywords:** TAVI, Thrombocytopenia, Platelet cell death, Phagocytic monocytes, Inflammatory cytokines

## Abstract

Thrombocytopenia after TAVI is common and clinically detrimental. Retrospectively, we observed Portico recipients had a more profound platelet drop than Evolut recipients. We thus investigated periprocedural platelet damage and/orpro-inflammatory state in 64 TAVI recipients at baseline and after implantation. Platelet damage was assessed by annexin V staining and monocyte-phagocytic phenotype was assessed according to CD14/CD36 expression. Serum cytokines were measured in 20 patients. The formaldehyde-based storage solution altered platelets. When, before being loaded onto the delivery system, Portico underwent one additional flushing to those recommended, the receiving patients showed thrombocytopenia, platelet damage, and CD36-monocyte count were mitigated. A general increase in IL-6 was recorded in overall TAVI recipients, but a high serum level of IL-8, a potent thrombocytopenia inducer, was measured in Portico recipients only, including those with extra-rinsed valve. Our study suggests a platelet-injury effect by storage-solution and generates the hypothesis of a role for the biomaterial in stimulating innate-immunity. Larger prospective studies are needed.

**Supplementary Information:**

The online version contains supplementary material available at 10.1007/s12265-021-10117-9.

**Figure:**
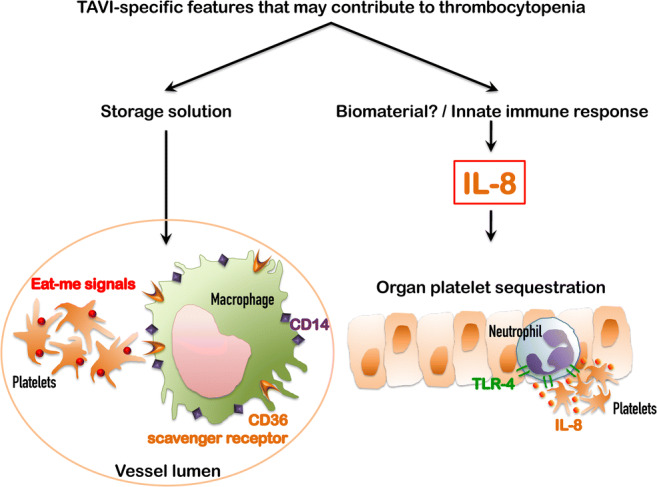
Graphical Abstract

## Introduction

Transcatheter aortic valve implantation (TAVI) is a method of treatment for severe aortic valve stenosis in patients with high/intermediate surgical risk. Consistent technical advances and simplification of the procedure are continuously underway, including miniaturization of delivery systems and improved biocompatibility of prostheses [1]. Indeed, latest generation TAVI devices, including Evolut (Medtronic, Minneapolis, MN, USA) and Portico (Abbott Vascular, Santa Clara, CA, USA), have incorporated refined features to reduce delivery catheter profile, facilitate deployment, and enable repositioning and retrieval capability, as well as valve durability. In view of the last 15 years advances, TAVI is expected to be ever more widely used [2]. Yet, among TAVI periprocedural complications, thrombocytopenia is still frequently occurring and insufficiently explained [3–9].

Severe thrombocytopenia (i.e., a platelet count < 50,000/ml) is luckily rarer [3, 10]. Yet, mild or moderate thrombocytopenia (i.e., a platelet count < 100,000/ml) is quite common and still associated even after multivariable adjustment with increased early and overall mortality after TAVI (e.g., due to fatal hemorrhage), as well as morbidity (e.g., hemorrhagic stroke or arterial bleeding) [3–10]. Notably, even nuisance thrombocytopenia may cost substantial resources by increasing hospital stay [10]. Several general mechanisms to explain post-TAVI thrombocytopenia have been proposed, including drug toxicity (e.g., heparin, aspirin, clopidogrel, warfarin, and novel oral anticoagulants), thromboinflammation, increased platelet consumption, mechanical damage due to shear stress (e.g., in case of paravalvular leak), activation of the coagulation cascade, decreased platelet production, and impaired platelet renewal, as well as dilution pseudothrombocytopenia [4, 11–15]. Further understanding the causes of thrombocytopenia is urgently needed to improve next-generation devices.

Expanding on anedoctic experience of a differential risk of thrombocytopenia in patients undergoing TAVI with the Portico and Evolut devices (i.e., higher risk with Portico) [16], we retrospectively appraised the occurrence of post-TAVI thrombocytopenia in a single large-volume tertiary center. Furthermore, we attempted to investigate the underlying mechanisms involved in the different impairment of platelet counts of Portico recipients (#P) and Evolut recipients (#E), by means of several detailed mechanistic experiments.

## Methods

### Patients

Patients undergoing TAVI at Pineta Grande Hospital, Castel Volturno, Italy, and providing written informed consent were enrolled as part of the prospective multicenter observational RISPEVA (Registro Italiano GISE sull’impianto di Valvola Aortica Percutanea), without any additional selection criterion [7, 17]. The study was approved by the competent ethical committee (approval number: 84/2010/VE). We retrospectively analyzed platelet counts of 76 consecutive patients (49 F and 27 M; mean age 82 ± 5) treated with Portico (Abbott Vascular) and 98 consecutive patients (57 F and 40 M; mean age 82 ± 6) treated with Evolut (Medtronic) in the period between February 2017 and February 2019. In light of a more remarkable thrombocytopenia in the Portico recipients, in the above described cohort, between March 2019 and February 2020, from the collected samples of additional 64 consecutive Evolut and Portico recipients treated at the same site, further analyses were performed. After cell counting [18], blood samples were fractionated in serum, platelets, and PBMCs. More precisely, blood was collected in vacutainer EDTA tubes from fasting patients, as part of routine management of the patient, starting the day before TAVI (T0). In detail, this second cohort included 20 patients (14 F and 6 M; mean age 82 ± 6) treated with Portico, 24 patients (15 F and 9 M; mean age 80 ± 5) with Evolut, and 20 patients (12 F and 8 M; mean age 80 ± 6) implanted with Portico prostheses undergoing an extra-rinsing, besides those recommended by the manufacturer (wPortico). Baseline and procedural features of the 2 cohorts are described in Tables S1 and S2, including baseline and discharge antithrombotic therapy, whereas device features are summarized in Table S3. Notably, periprocedural antithrombotic therapy entailed in all patients weight adjusted unfractioned heparin (target activated clotting time 200-300 s). Informed consent was obtained from each patient and the study protocol conforms to the ethical guidelines of the 1975 Declaration of Helsinki as reflected in a priori approval by the institution’s human research committee.

Clinical information and the results of the study were handled by authorized personnel only. In compliance with patients’ rights, patient identity was kept confidential.

### Platelet-rich Plasma Preparation and Peripheral Blood Mononuclear Cells Isolation

Platelet-rich plasma (PRP) and peripheral blood mononuclear cells (PBMCs) were isolated from 24 Evolut recipients and 40 Portico recipients (20 #P + 20 #wP). PRP was obtained from 5 ml blood collected in sterile K_3_EDTA vacutainer tubes by centrifugation at 180×*g* for 10 min. The supernatant, i.e., PRP, was then further centrifuged at 4000 rpm for 4 min at room temperature, and the pellet containing platelets was then processed for analysis by immunofluorescence and flow cytometry. When needed, a lysis buffer for red blood cells (1X RBC Lysis Buffer, eBioscience, Invitrogen, Carlsbad, CA, USA) was added to platelets and incubated on a shaker for 10 min at room temperature. After removal of PRP, the corpuscular blood component was diluted 1:3 with isotonic saline solution and stratified on Ficoll-Hypaque density gradient (Histopaque-1077®, Sigma-Aldrich, St. Louis, MO, USA) to obtain PBMC. After a twice washing, PBMCs were resuspended in 5% FBS-RPMI 1640 (Biowest, Nuaillè, France), counted, and stained for immunofluorescence. For experiments with valve discard fluids, PRP from 3 normal donors was incubated (1:1, v/v) with valve discard fluid, or isotonic saline solution as control, for 8 h at 37 °C in a 5% CO_2_ humidified atmosphere. Then, PRP pellet was processed for platelet apoptosis assay (see “Flow Cytometry Analysis”).

### Flow Cytometry Analysis

BD-Pharmigen Fc block (2.5 μg/10^6^ cells) (Pharmingen/Becton Dickinson BD, San Diego, CA, USA) was used to minimize non-specific binding of immunoglobulins to Fc receptors, prior to the immuno-staining.

Platelets apoptosis assay was performed by annexin V staining in multiple fluorescence with mouse monoclonal antibodies recognizing the following typical cluster differentiation (CD): CD45-peridinin chlorophyll protein complex (PerCP)-, CD42b-phycoerythrin (PE)-, and CD61-fluorescein isothiocyanate (FITC)-conjugated antibodies (HI30, HIP1, CLB-thromb/1 C17clones, respectively; Immunotools, Friesoythe, Germany). Briefly, 1 × 10^5^ cells were extracted from 5 ml heparinized blood and resuspended in 100 μl of binding buffer (10 μM Hepes/NaOH pH 7.5, 140 μM NaCl, and 2.5 μM CaCl_2_) containing 1 μl of annexin V-allophycocyanin (APC)-conjugated (Pharmingen/Becton Dickinson BD) and 5 μl of each above-mentioned antibodies for 15 min at room temperature in the dark. Then, 100 μl of the same buffer was added to each sample and analyzed with BD Accuri^TM^ C6 Cytometer (Becton Dickinson BD). Platelets gating was performed on CD45^dim^/CD42b^+^/CD61^+^ cells. Macrophages were assessed by a multiple immunofluorescence staining of PBMCs with 5-10 μl of mouse monoclonal antibodies against CD45-PerCP-, CD14-APC- (TÜK4 clone; MiltenyiBiotec, BergischGladbach, Germany), CD36-FITC- (NL07 clone; eBioscience, Invitrogen), and anti-HLA-DR-PE- (L243 clone, eBioscience, Invitrogen) conjugated antibodies, added to 50 μl of PBMC suspension. Cells were incubated for 15 min in the dark at room temperature (20-25 °C). For each staining, a relative Ig isotype conjugated antibody was used as a control of non-specific binding. Monocyte gating and subset counts were performed on CD45^+^/CD14^+^ cells. Samples were acquired using the BD Accuri^TM^ C6 Cytometer, as previously described. The flow cytometry data were analyzed with the FlowJo software or the C6 Accurì software.

### Cytokine Profile

Sera from the first 7 #E and 13 Portico recipients (7# P + 6 #wP) from the second cohort were screened for the concentration of IL-2, IL-4, IL-6, IL-8, IL-10, GM-CSF, IFN-γ, and TNF-α, using the Bio-Plex(8-plex) multiplex Human Cytokine and Growth factor assay kit (Bio-Rad, Hercules, CA, USA) according to the manufacturer’s instruction. Briefly, sera were centrifuged at 1000×*g*, diluted 1:4, and then run along with standards and control samples. The assay was performed in the 96-well filtration plate supplied with the Bio-Plex kit. Premixed fluorescent-labeled microspheres, coated with target antibodies, were used and the samples were read using a Luminex MAGPIX instrumentation. Data were analyzed using the Bio-Plex Manager software and standards served to determine the unknown cytokine concentration automatically calculated with a 5PL curve fitting by creating a standard curve from a serial dilution of standards to 8 concentrations ranging from 32,000 to 1.95 pg/ml. Cytokine concentrations in patients’ sera were measured at different times after the procedure and expressed as fold increase compared to the baseline (T0) level of each patient.

### Statistical Analysis

Student’s *t*-test was used to analyze the differences between the means of values. For multiple comparisons, ordinary one-way ANOVA was performed using GraphPad Prism 7.0a Macintosh. *P* value ≤ 0.05 was considered statistically significant.

## Results

### In Comparison with #E, #P Exhibit Increased Platelet Cell Death

**Fig. 1:**
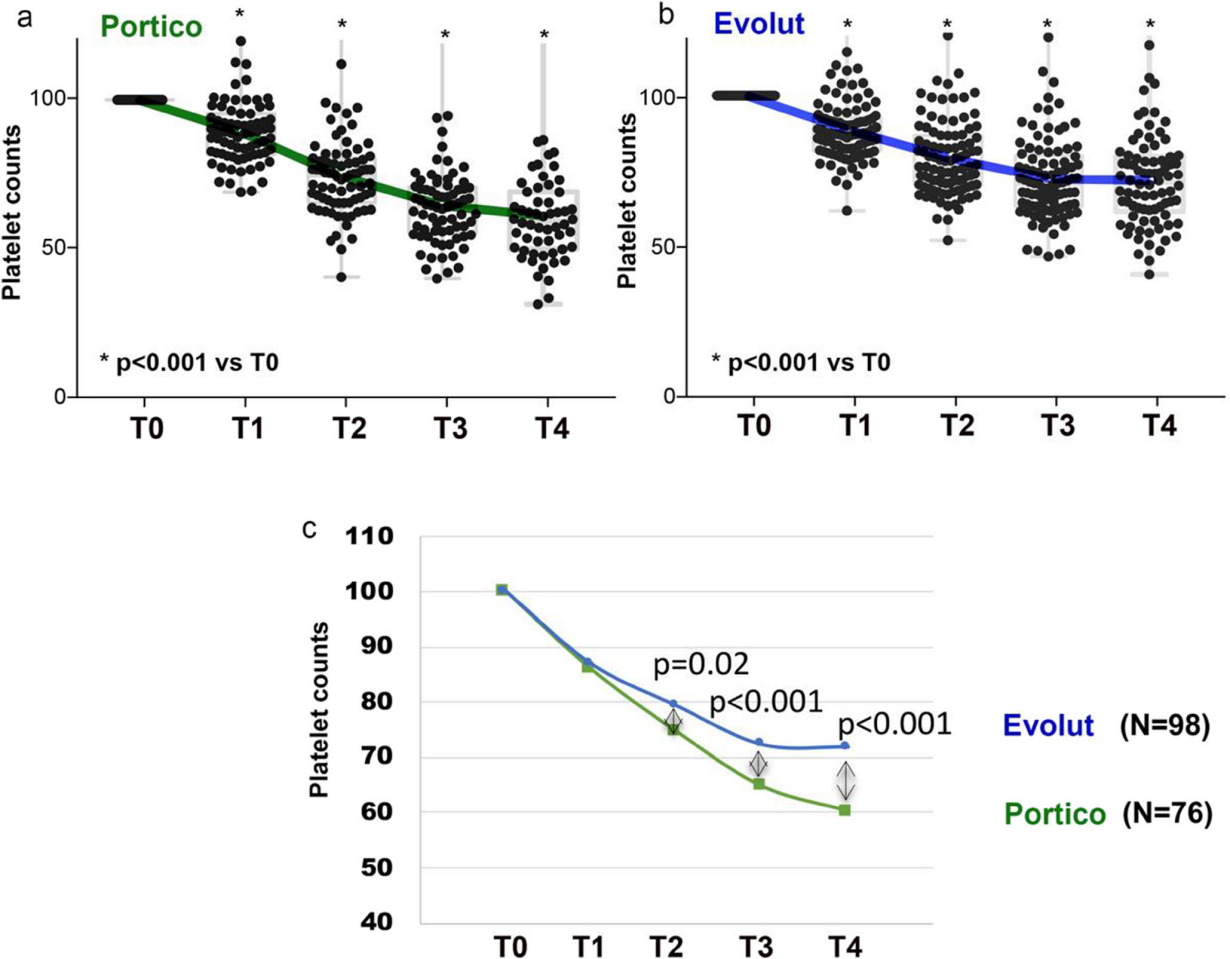
Platelet counts from 76 Portico and 98 Evolut recipients (retrospective analysis). Values were taken at hospitalization (T0), 2-4 h after implantation (T1), and after 1, 2, and 3 days from implantation (T2, T3, T4). Graphs in (**a**) and (**b**) show the platelet count from 76 Portico patients (**a**, *N* = 76 biological replicates) and 98 patients implanted with Evolut (**b**, *N* = 98 biological replicates); each dot corresponds to a single observation/patient (*N* = 1 technical replicates) taken at each indicated time (values were normalized to 100 [T0]). In each graph, Dunnett’s multiple comparisons test was used to assess changes (*) vs T0 of platelet counts after TAVI. **c** Curves of platelet counts in patients implanted with Portico and Evolut. Plotted values are means of values represented in **a** green line, *N* = 76 biological replicates/each time point, and **b** blue line, 98 biological replicates/each time point. Comparison between values was done at each T0-T4 time, using Student’s *t*-test

**Fig. 2:**
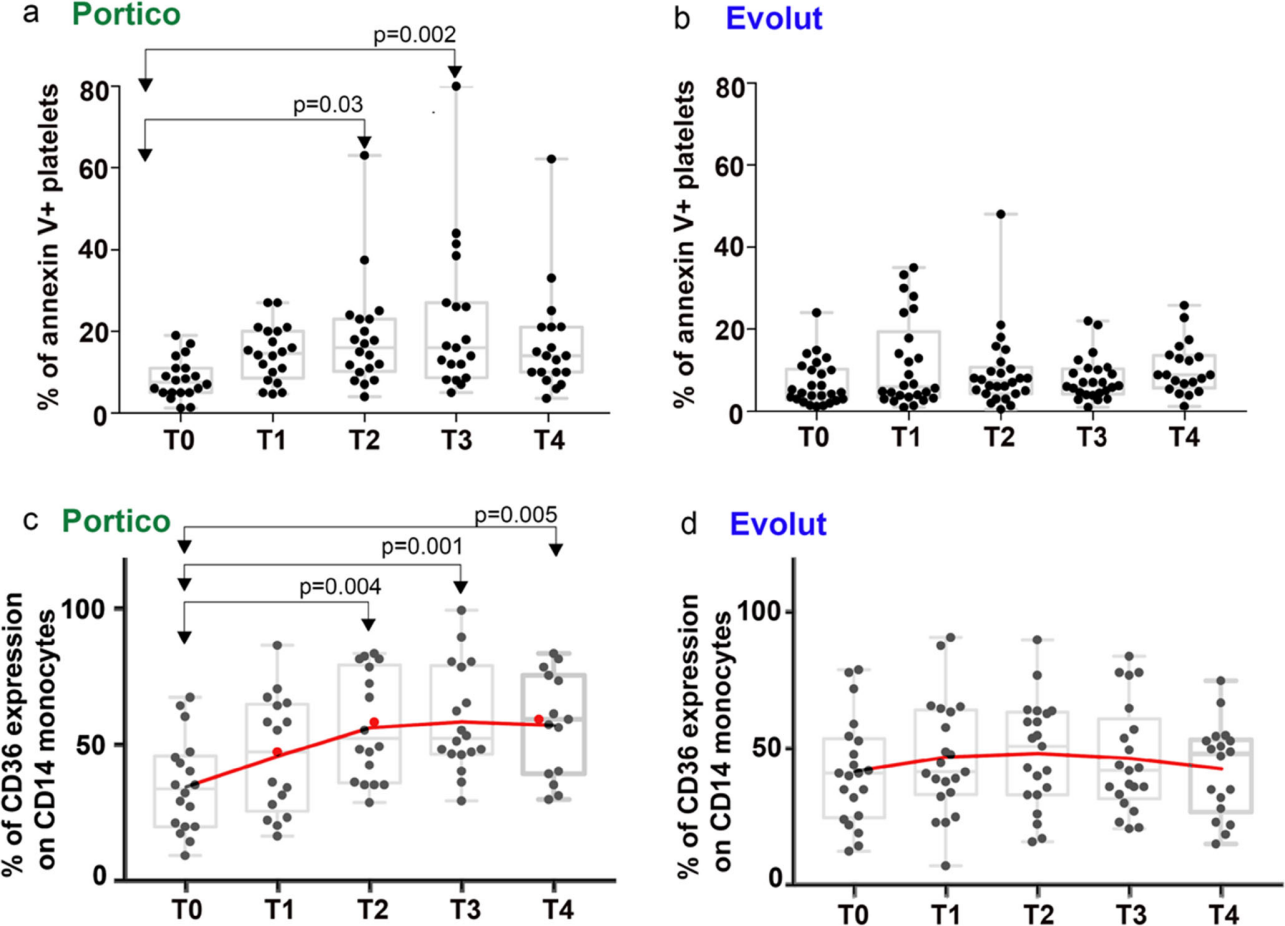
Analysis of platelet damage and monocyte phagocytic phenotype in peripheral blood of Portico and Evolut recipients. Platelet damage was assessed by annexin V staining. Graphs in (**a**) and (**b**) show the percentage of annexin V^+^ platelets from peripheral blood of 20 #P and 24 #E in (**a**, *N* = 20 biological replicates) and (**b**, *N* = 24 biological replicates), respectively. Graphs in (**c**) and (**d**) show the percentage of CD36^+^ monocytes from peripheral blood of #P (*N* = 20 biological replicates) and #E (*N* = 24 biological replicates). Overall values were taken at hospitalization (T0), after 2-4 h after implantation (T1), and after 1, 2, and 3 days from implantation (T2, T3, T4). In each graph, comparison of T1–T4 values vs T0 was performed using Dunnett’s multiple comparisons test

We retrospectively analyzed platelet levels of 76 #P and 98 #E treated between February 2017 and February 2019. Baseline characteristics and procedural features of the 2 groups are described in Table S1. The analysis was conducted at hospitalization (T0), after 2-4 h from implantation (T1), and 1, 2, and 3 days after implantation (T2, T3, T4, respectively). Values (normalized to 100) at each time point were significantly reduced (*P* < 0.001) compared to T0 in both patients’ groups (#P: T0 100, T1 85.9 ± 20.8, T2 74.5 ± 14.0, T3 64.7 ± 14.9, T4 60.2 ± 19.6; #E: T0 100, T1 86.9 ± 17.2, T2 79.3 ± 13.2, T3 72.3 ± 14.3, T4 71.8 ± 16.2) (Fig. 1a, b). However, post-TAVI normalized platelet counts in #P were significantly lower than those of #E from T2 to T4, suggesting a greater impact of Portico implantation on thrombocytopenia (Fig. 1c). To elucidate the possible mechanisms underlying this difference between the two devices, we measured the extent of platelet death and the expression level of CD36 on monocytes in peripheral blood; these measurements were made in the second study cohort consisting of 20 patients #P, 24 #E, and 20 #wP, whose baseline characteristics and procedural features are described in Table S2. A workflow of our study is depicted in Fig. S1. We hypothesized, in the case of platelet damage, the upregulation of CD36 as this receptor is involved in the endocytic uptake of altered self-components, including oxidized phospholipids and PS [19]. Data from 20 #P and 24 #E of this second cohort confirmed the same result in terms of differential between devices drop of platelet counts already showed in the previous cohort (Fig. S2). To assess PS externalization on the platelet plasma membrane, we used annexin V staining and flow cytometry. Precisely, platelet samples were stained with CD45/CD42b/CD61/AnnexinV: CD45 served to exclude eventual mononuclear cells (CD45^bright^) from analysis, while CD42b and CD61 are specific platelet markers. To assess phagocytes, monocytes were stained with CD45/HLA-DR/CD36/CD14. Fig. 2 shows levels of annexin V^+^ platelets and CD36 expression in monocytes from #P (Fig. 2a, b) and #E (Fig. 2c, d). In #P, compared to basal value, the level of annexin V^+^ platelets was significantly increased at T2 and T3 (Fig. 2a). In #E, no significant increase in platelet death was registered (Fig. 2b). In line with platelet damage, CD36^+^ monocytes were increased from T2 to T4 in #P (Fig. 2c), but not in #E (Fig. 2d). A representative flow cytometry assessment of platelet damage and CD36^+^ monocytes in a patient implanted with Portico is shown as Fig. S3. In the upper histograms CD61^dim^/CD42b^dim^ (gated platelets) are consistent with injured platelets that increase after TAVI. Similarly, annexin V^+^ platelets (intermediate histograms) and CD36^+^ monocytes (lower histograms) progressively increase after TAVI.

### The Bioprosthesis Storage Solution Induces “Eat Me” Signals on Platelet Plasma Membrane

**Fig. 3:**
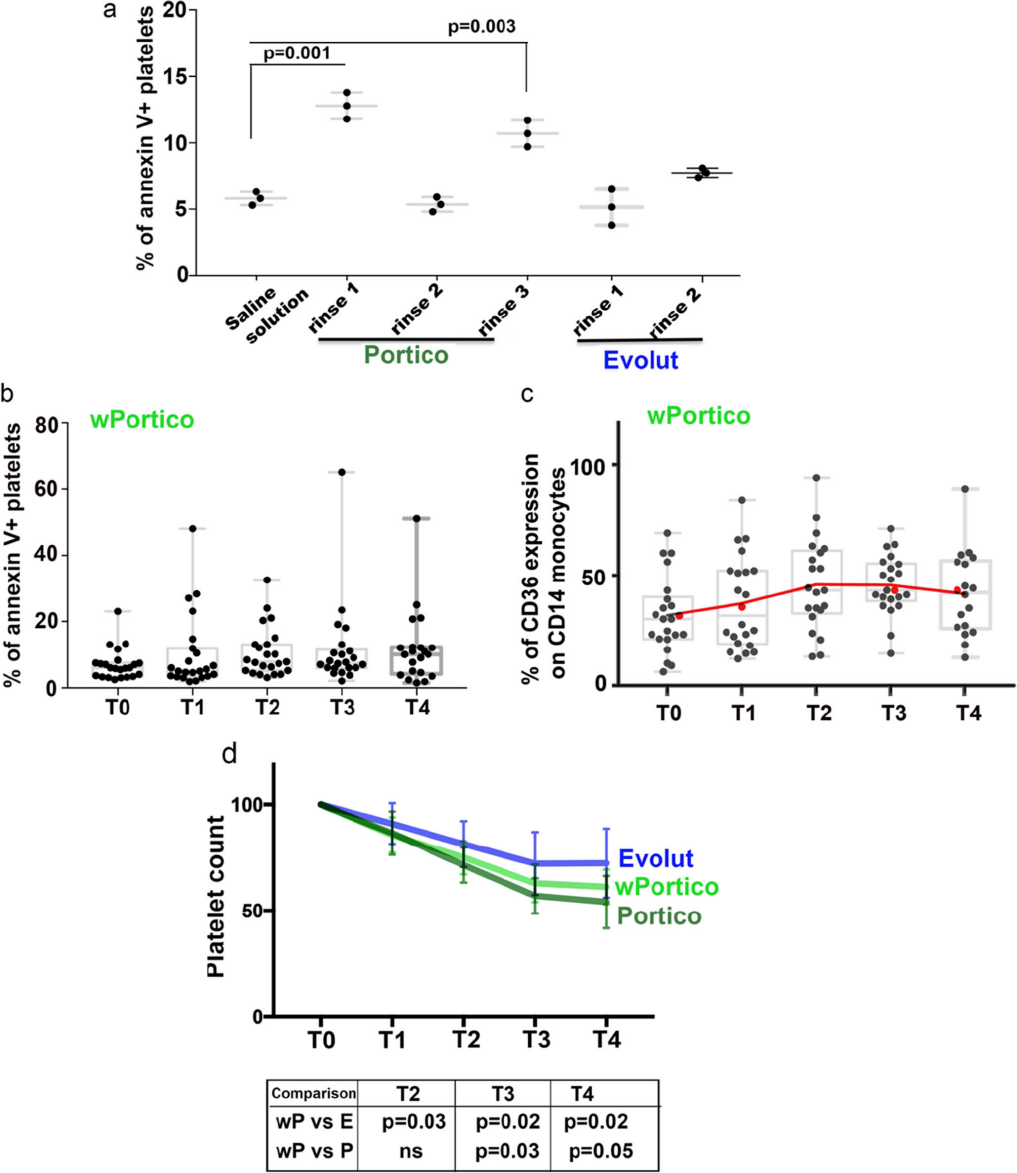
Effect of bioprosthesis storage solution on platelet cell death. **a** Effect of Portico and Evolut discard fluids (respectively, 3 and 2 consecutive valve rinses) on expression of eat-me signals on platelet plasma membrane. Graph shows the percentage of annexin V^+^ platelets from 3 healthy donors (*N* = 3 biological replicates) that were incubated with discard fluids for 8 h. Each dot corresponds to the mean of technical triplicates (*N* = 3) for each healthy donor. Comparison between values obtained with saline solution and those obtained with discard fluids was done using Student’s *t***-**test. Graphs in (**b**) and (**c**) show the percentage of annexin V^+^ platelets and values of CD36^+^ monocytes, respectively, from *N* = 20 #wP biological replicates, taken at T0-T4. In each graph, Dunnett’s multiple comparisons test did not show any difference between T1-T4 values vs T0. **d** Curves of platelet counts in patients implanted with Portico, Evolut, and wPortico. Plotted values are means ± SD of platelet counts from *N* = 24 #E (blue line), *N* = 20 #P (green line), and *N* = 20 #wP (light green line) biological replicates. Platelet counts (normalized to 100 [T0]) were taken at T0–T4. Comparison between patient groups was done at each time point using Student’s *t*-test

Portico and Evolut bioprostheses are packaged in formaldehyde and glutaraldehyde storage solutions, respectively. According to instructions for use, Portico requires 2 rinses in 2 different bowls with a sterile isotonic saline solution while Evolut a single rinse in sterile isotonic saline solution. To address whether the storage solutions can alter platelet membranes, we investigated the effect of the valve-discarded fluids on platelets isolated from 3 normal donors, using the saline solution as a control. Both valves received an extra-rinse to those recommended (3 and 2 rinses for Portico and Evolut, respectively). As shown in Fig. 3a after an 8 h incubation, Portico-rinse 1 induced a significant PS externalization on the platelet plasma membrane. The same effect appeared to be reduced with rinse 2 but re-appeared with rinse 3, suggesting that the fixative continues to be released after an apparent complete removal. Both discard fluids from Evolut did not show a significant effect on PS externalization of platelets’ plasma membrane, even if the second discard fluid appeared to have a slight effect. To address whether Portico-induced thrombocytopenia could be mitigated by more effective removal of the fixative, an additional rinse of the bioprosthesis was regularly performed before TAVI implantation on 20 patients (#wP). Analysis of platelet death and CD36^+^ monocytes showed that similarly to #E, #wP did not significantly change these values after TAVI (Fig. 3b, c). Analysis of platelet counts showed that platelet drop was significantly relieved in #wP (Fig. 3d); however, the platelet counts of #wP remained significantly lower than those of #E (Fig. 3d). Collectively, these results suggest a role for formaldehyde in thrombocytopenia of #P.

### Different Cytokine Profile in Patients Implanted with Portico, Evolut, or wPortico

**Fig. 4:**
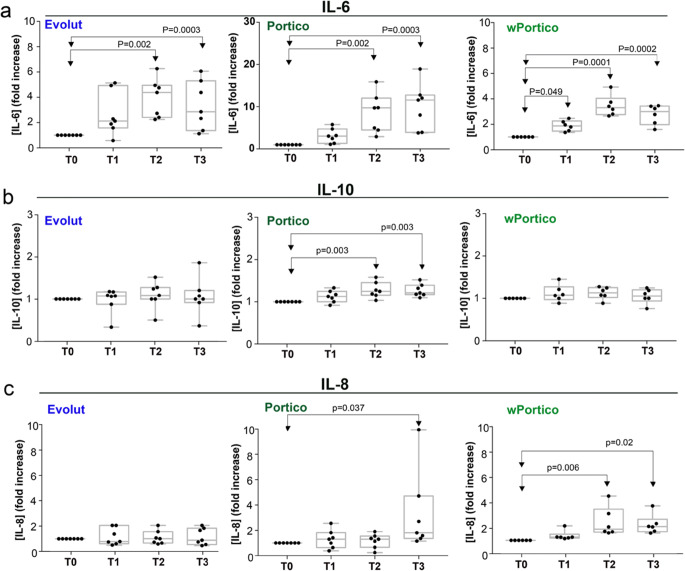
Cytokine levels in sera of patients implanted with Portico, Evolut, and wPortico. Levels of IL-6 (**a**), IL-10 (**b**), and IL-8 (**c**) at T0–T3 are from 7 #P (*N* = 7 biological replicates), 7 #E (*N* = 7 biological replicates), and 6 #wP (*N* = 6 biological replicates). Each dot (biological replicate) corresponds to the mean value of technical duplicates (*N* = 2) from a single serum sample. Values are expressed as fold increase compared to the baseline (T0). In each graph, Dunnett’s multiple comparisons test was performed to compare T1–T3 values vs T0

Previous authors have reported a systemic inflammatory response to TAVI implantation with increased levels of interleukin (IL)-6 and IL-8 [4, 5]. We analyzed the cytokine profile of patients’ sera using a multi-cytokine assay for IL-6, IL-8, IL-10, IFN-γ, IL-2, IL-4, TNF-α, and GM-CSF. Sera samples were obtained at T0-T3 from 7 #P, 7 #E, and 6 #wP. We could not measure significant variations in IFN-γ, IL-2, IL-4, TNF-α, and GM-CSF levels in patient sera before and after TAVI (Fig. S4). Alterations in IL-6, IL-8, and IL-10 levels were instead registered. More precisely, IL-6 resulted in a significant increase at T2 and T3, compared to the baseline (T0)(Fig. 4a) in all TAVI recipients. An increase in IL-6 was also registered at T1, although it was significant only in #wP. Notably, the increase was more pronounced in #P (Fig. S5) than other TAVI recipients. IL-10 resulted in an increase at T2-T3, compared to the baseline (T0)(Fig. 4b) in #P only. IL-8 was increased in #P at T3, and earlier, at T2 and T3 in #wP (Fig. 4c).

## Discussion

The causes of thrombocytopenia after TAVI remain poorly understood [11]. Herein, based on the observation of a more profound thrombocytopenia in patients receiving Portico valve than in Evolut recipients, we investigated the mechanisms underlying such a diversity, in an attempt to provide insights into the pathogenetic factors of platelet drop after TAVI.

Previous authors have described a systemic inflammatory response syndrome in patients receiving TAVI [20, 21]. Such an inflammatory state concurs to thrombocytopenia in recipients [22]. Periprocedural platelet damage also contributes to thrombocytopenia [23]. To investigate platelet injury, we measured the exposure of phosphatidyl-serine(PS) on the plasma membrane, which is an “eat me” signal for phagocytes. At the same time, because phagocytosis of altered or senescent platelets is the primary mechanism of platelet clearance, we measured the expression of CD36 on peripheral monocytes to assess whether a phagocytic asset accounted for platelet removal. As known, professional phagocytes exploit archetypal pattern recognition receptors. Among these receptors, CD36 plays a relevant role because it binds polyanionic ligands of both pathogen and self-origin [24]. Moreover, we measured serum levels of pro-inflammatory cytokines like interleukin (IL)-6 and IL-8, to address the possible co-existence of a pro-inflammatory state.

Our findings show that #P platelets expressed eat-me signals. Concordantly, the counts of CD36^+^ monocytes were increased in #P, but #E, as soon as after 24 h from TAVI implantation. We also found that the storage solution of Portico bioprosthesis produced an increased PS-exposure on the plasma membrane of control donors’ platelets. When, before being loaded onto the delivery system, Portico underwent one additional flushing to those recommended (washed Portico), the receiving patients (#wP) showed thrombocytopenia, platelet damage, and CD36-monocyte count were mitigated. Regarding serum cytokines, IL-8, differently from IL-6 that was increased in overall TAVI recipients, was upregulated in #P and #wP, but #E. IL-8 is secreted by any cells with toll-like receptors that are involved in the innate immune response [25]. This cytokine is a potent platelet-activating factor [26] and promotes thrombocytopenia [27]. Given the different biomaterial of the two prostheses (bovine for Portico vs porcine for Evolut), it could be hypothesized a role for the biomaterial type in eliciting an innate immune response.

Accordingly, our study suggests a role for Portico storage solution in platelet damage. In #P, platelet injury was testified by exposure of eat-me signals on platelet plasma membrane which was associated with an increase in CD36^+^ monocytes, consistent with phagocytosis stimulation. CD36 is a key regulator of IL-10 expression, in turn IL-10 stimulates phagocytosis [28]. In accordance with this finding, IL-10 increase was registered in #P, but #E. After a more accurate removal of the fixative agent from the Portico valve before implantation, no increase in annexin V^+^ platelets, CD36^+^ monocytes, and IL-10 was registered in recipients, and platelet drop was also relieved. However, platelet levels in #wP remained still lower than those in #E. Assessment of cytokine levels showed that TAVI implantation produced a sustained increase of IL-6 in patient sera, in accordance with previous studies [20, 21], even if a more remarkable increase was registered in #P patients. Platelet activation is a potent IL-6 inducer [29]. Even if it was significant only in #wP, an increase in IL-6 level was registered very early after TAVI implantation, possibly due to periprocedural platelet activation. Regarding IL-8, only patients receiving Portico showed an increase in this cytokine level. Unexpectedly, such an increase occurred earlier in #wP than #P. As known, IL-8 is secreted by macrophages that are involved in the innate immune response [25], raising the hypothesis of a role for the biocomponent of the prosthesis in inducing the secretion of IL-8. In #wP, an early unmasking of the heterologous biocomponent of the valve, possibly due to the more accurate removal of the fixative, could account for the earlier IL-8 upregulation. Macrophages are the first cells to release IL-8 to recruit other cells [25]. During inflammation, IL-8 levels, along with high expression levels of toll-like receptor 4 (TLR4) on macrophages and platelets [30], are reported to contribute to thrombocytopenia through neutrophil-dependent pulmonary sequestration [27]. Larger prospective studies are needed to confirm the role of IL-8 in TAVI thrombocytopenia.

Our findings thus support the hypothesis that the fixative, i.e., formaldehyde, contained in the storage solution of some TAVI prostheses can cause platelet injury and concurs, at least in part, to thrombocytopenia. This effect is avoided by extra rinsing of the valve in addition to those recommended. Other mechanisms are likely involved, including an innate immune response to specific bioprosthesis components. The study is clearly not powered for clinical outcomes evaluation and post-TAVI thrombocytopenia could possibly recognize several pathogenetic mechanisms [31] of which our study has highlighted only a few. However, it is reasonable to hypothesize that particular clinical conditions, such as hematologic diseases or coagulation disorders, may in the future guide the choice of the valve prosthesis. Indeed, there is ample evidence that thrombocytopenia after TAVI may have substantial economic implications (e.g., by prolonging hospital stay) but most importantly may adversely impact on morbidity (e.g., by increasing the risk of major bleeding or vascular complications) and mortality (e.g., by increasing the risk of fatal bleeding) [5–8], and this holds even truer in patients considered for TAVI, who are typically at intermediate, high, or prohibitive surgical risk.

The present analysis could not avoid certain limitations. First, given the non-randomized nature of the registry, data would result in selection bias, even though patients were consecutively enrolled from a high-volume center. Second, the choice of device type, procedural strategy, and periprocedural management were left to the physician’s discretion. Accordingly, our findings should mainly be regarded as hypotheses-generating and require further confirmation from a large pragmatic randomized trial.

## Supplementary Information

ESM 1 (PDF 2357 kb).

## Abbreviations

TAVI: Transcatheter aortic valve implantation
#P: Portico recipients
#E: Evolut recipients
PS: Phosphatidyl-serine
IL: Interleukin
#wP: Washed Portico
RISPEVA: Registro Italiano GISE sull’impianto di Valvola Aortica Percutanea
PRP: Platelet-rich plasma
PBMCs: Peripheral blood mononuclear cells
RBC: Red blood cells
CD: Cluster differentiation
PerCP: Peridinin chlorophyll protein complex
PE: Phycoerythrin
FITC: Fluorescein isothiocyanate
APC: Allophycocyanin-conjugated
TLR4: Toll-like receptor 4

## Declarations

### Animal Studies

No animal studies were carried out by the authors for this article.
