# Supplementary material for: Thrombocytopenia Complicating Transcatheter Aortic Valve Implantation: Differences Between Two New-Generation Devices
Source: J Cardiovasc Transl Res. 2021 Mar 15;14(6):1104–13. doi: 10.1007/s12265-021-10117-9 (PMC8651580; doi:10.1007/s12265-021-10117-9)
Supplement: Supplementary file 1 — (PDF 2357 kb). [file 12265_2021_10117_MOESM1_ESM.pdf]

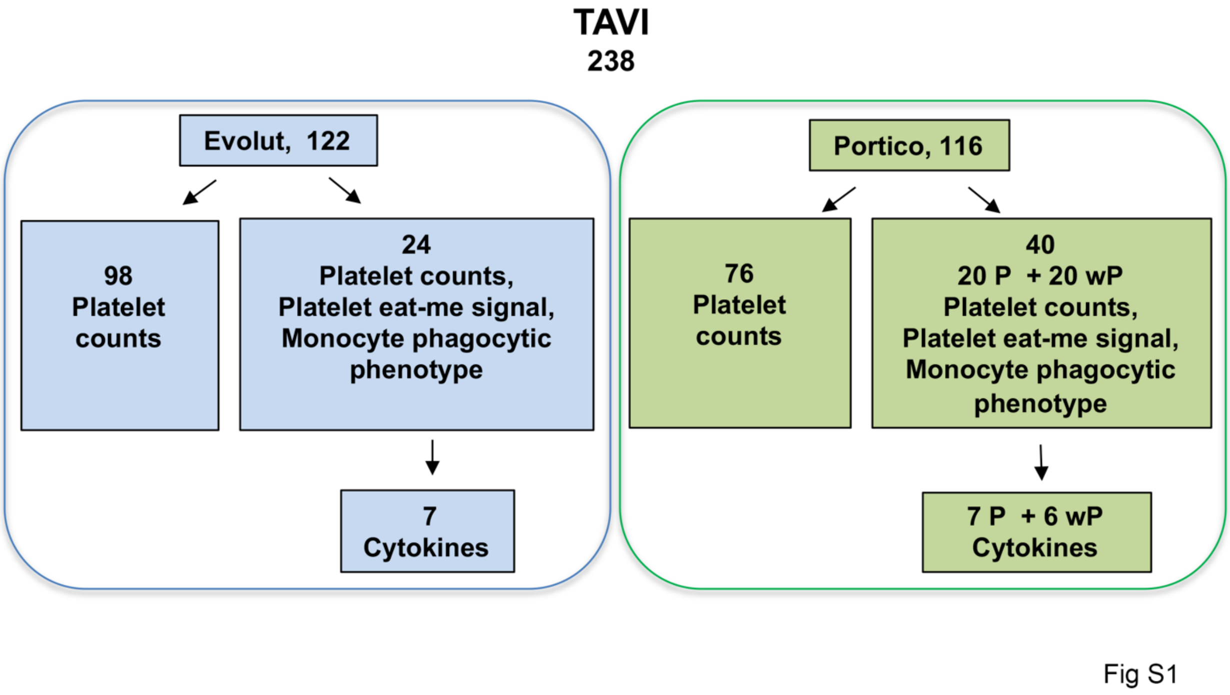

Fig. S1 *Workflow of the study*

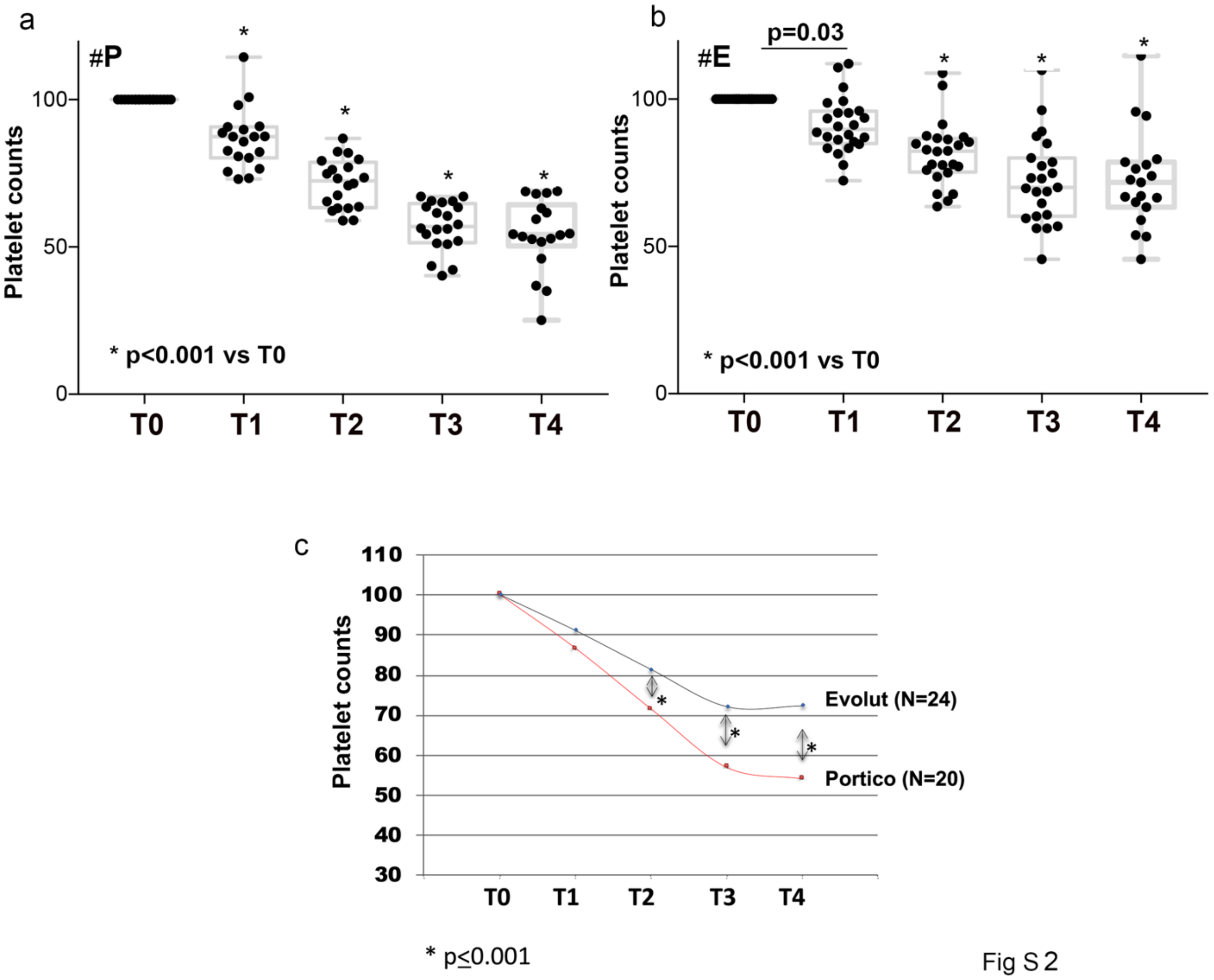

Fig. S2 *Platelet counts in Portico and Evolut recipients (prospective study)* Values were taken at hospitalization (T0), 2-4 h after implantation (T1) and after 1, 2, and 3 days from implantation (T2, T3, T4). Graphs in a and b show the platelet count from 20 Portico patients (a, N=20 biological replicates) and 24 patients implanted with Evolut (b, N=24 biological replicates); each dot corresponds to a single observation/patient (N=1 technical replicates) taken at each indicated time (values were normalized to 100 [T0]). In each graph, Dunnett's multiple comparisons test was used to assess changes (*) vs T0 of platelet counts after TAVI. (c) Curves of platelet counts in patients implanted with Portico and Evolut. Plotted values are means of values represented in (a, Portico, N=20 biological replicates) and (b, Evolut N=24 biological replicates). Comparison between values was done at each T0-T4 time, using Student T test.

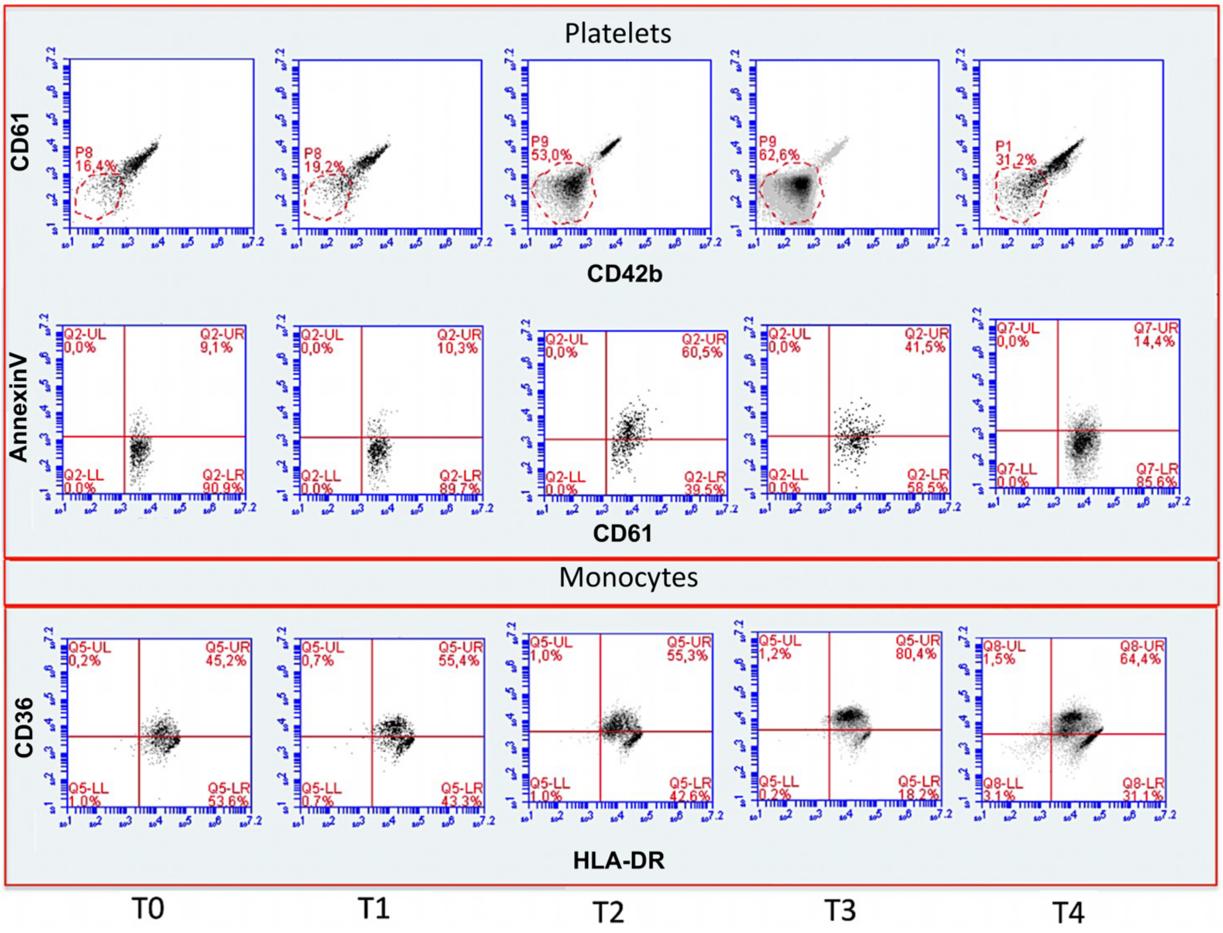

Fig S3

Fig. S3 *Flow cytometry assessment of platelet damage and CD36*$^{+}$ *monocytes in a patient implanted with Portico* In the upper histograms, CD61^{dim}/CD42b^{dim} (gated platelets) are consistent with injured platelets that increase after TAVI. Similarly, annexin V^{+} platelets (intermediate histograms) and CD36^{+} monocytes (lower histograms) progressively increase after TAVI.

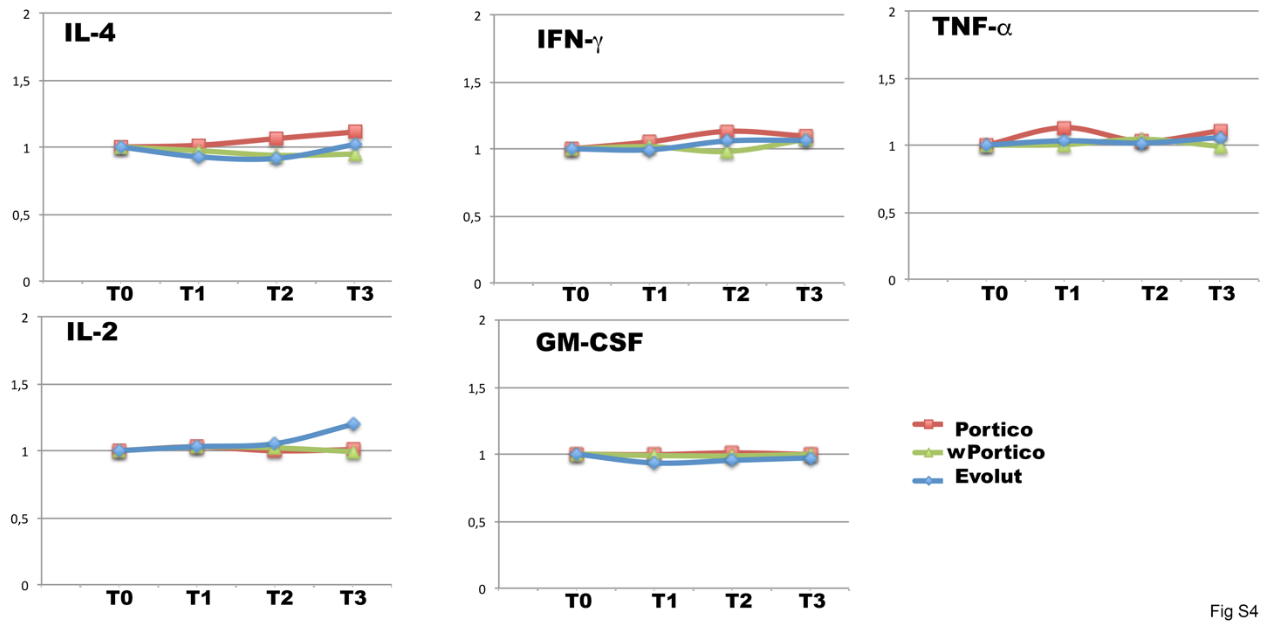

Fig. S4 *No significant variations in IFN-γ, IL-2, IL-4, TNF-α, GM-CSF levels in patient sera before and after TAVI Cytokine levels in sera of patients implanted with Portico, Evolut and wPortico*. Plotted values, for each curve, are means of biological replicates (Portico, N=7 biological replicates; Evolut, N=7 biological replicates; wPortico, N=6 biological replicates). Biological replicates, in turn, were means of technical duplicates from a single serum sample. Values are expressed as fold increase compared to the baseline (T0). In each graph, Dunnett's multiple comparisons test was performed to compare T1-T3 values vs T0. No significance was calculated.

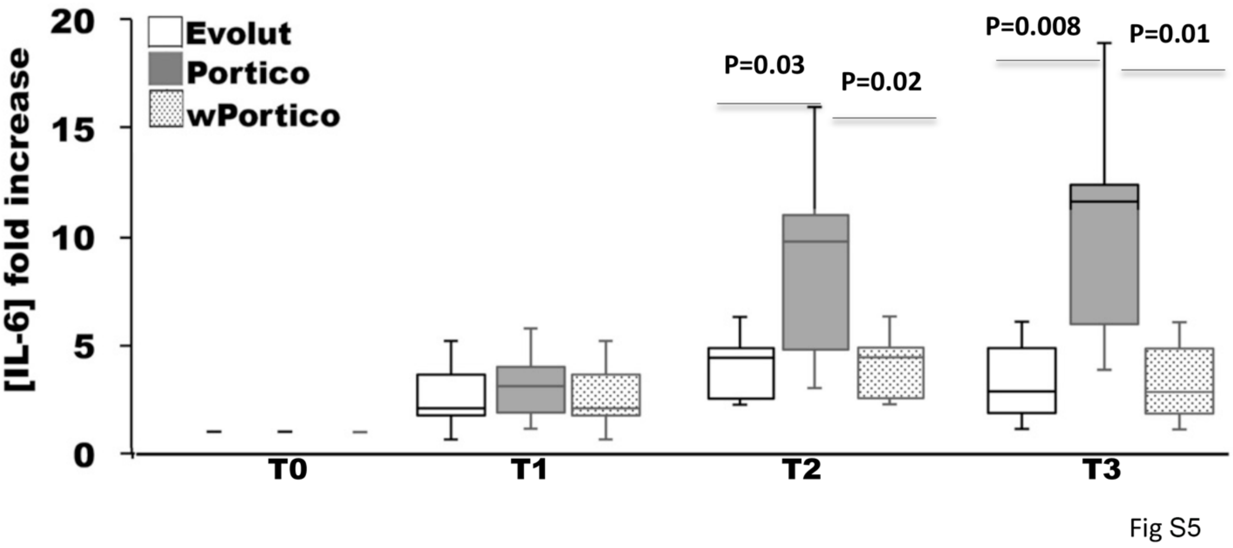

Fig. S5 *Comparison of IL-6 levels in TAVI recipients* IL-6 levels, measured at T0-T3, are represented as box plots of biological replicates from 7 #P, 7 #E, and 6 #wP. Each biological replicate, in turn, was the mean of technical duplicates from a single serum sample. Values are expressed as fold increase compared to the baseline (T0). Student T test served for comparison between groups.

Table S1. Baseline and procedural features of patients treated between February 2017 and September 2019 divided according to the implanted device.

	Overall (n=174)	***#E*** (n=98)	***#P*** (n=76)	***P value***
Baseline characteristics				
Age, years	81.6±5.5	81.6±5.9	81.7±4.9	0.905
Female sex	106 (60.9%)	57 (58.2%)	49 (64.5%)	0.397
Arterial hypertension	135 (77.6%)	75 (76.5%)	60 (78.9%)	0.705
Diabetes mellitus	39 (22.4%)	21 (21.4%)	18 (23.7%)	0.723
Atrial fibrillation	30 (19.2%)	14 (17.5%)	16 (21.1%)	0.685
eGFR, mL/min	64.3±20.0	66.4±20.7	61.5±18.8	0.110
Prior aortic valvuloplasty	8 (4.6%)	5 (5.1%)	3 (3.9%)	1.000
Severe chronic kidney disease*	32 (18.4%)	21 (21.4%)	11 (14.5%)	0.240
Chronic obstructive pulmonary disease	48 (27.6%)	29 (29.6%)	19 (25.0%)	0.501
Left ventricular ejection fraction, %	51.3±8.3	50.9±8.4	51.9±8.1	0.439
Mean aortic gradient, mmHg	52.4±18.0	52.7±19.8	52.0±15.5	0.805
Peak velocity, cm/m^2	4.6±0.6	4.6±0.7	4.6±0.6	0.848
Baseline therapy				
Aspirin	82 (60.7%)	31 (54.4%)	51 (65.4%)	0.215
Clopidogrel	40 (29.6%)	16 (28.1%)	24 (30.8%)	0.849
Warfarin	3 (2.2%)	1 (1.8%)	2 (2.6%)	1.000
Novel oral anticoagulant	23 (17.0%)	12 (21.1%)	11 (14.1%)	0.356
Dual antiplatelet therapy	32 (23.7%)	13 (22.8%)	19 (24.4%)	1.000
Single antiplatelet agent plus anticoagulant	5 (3.7%)	2 (3.5%)	3 (3.9%)	1.000
Triple antithrombotic therapy	0	0	0	1.000
Procedural features				
Femoral access	168 (96.6%)	95 (96.9%)	73 (96.1%)	0.751
Closure device use	169 (97.1%)	97 (99.0%)	72 (94.7%)	0.097
Postdilation	93 (53.4%)	57 (58.2%)	36 (47.4%)	0.157
Postprocedural paravalvular regurgitation**				0.137
None	79/141 (56.0%)	44/80 (55.0%)	35/61 (57.4%)	0.778
Mild	57/141 (40.4%)	31/80 (38.8%)	26/61 (42.6%)	0.642
Moderate	5/141 (3.5%)	5/80 (6.3%)	0/61 (0.0%)	0.069
Discharge features				
Aspirin	138 (85.7%)	66 (80.5%)	72 (91.1%)	0.071

Clopidogrel	118 (73.3%)	62 (75.6%)	56 (70.9%)	0.594
Warfarin	13 (8.1%)	4 (4.9%)	9 (11.4%)	0.155
Novel oral anticoagulant	28 (17.4%)	15 (18.3%)	13 (16.5%)	0.837
Dual antiplatelet therapy	106 (65.8%)	53 (64.6%)	53 (67.1%)	0.868
Single antiplatelet agent plus anticoagulant	32 (19.9%)	13 (15.9%)	19 (24.1%)	0.237
Triple antithrombotic therapy	2 (1.1%)	1 (1.0%)	1 (1.3%)	1.000
Paravalvular regurgitation§				0.591
None	103/157 (65.6%)	51/81 (63.0%)	52/76 (68.4%)	0.472
Mild	49/157 (31.2%)	28/81 (34.6%)	21/76 (27.6%)	0.349
Moderate	2/157 (3.2%)	2/81 (2.5%)	3/76 (3.9%)	0.674

Values are expressed as mean±SD or n (%).
--#P= Portico recipients; #E= Evolut recipients.
eGFR= Estimated glomerular filtration rate.
*defined as eGFR<30 mL/min.; **data available for 141 patients; §data available for 157 patients.

Table S2. Baseline and procedural features of patients treated between September 2019 and February 2020 divided according to the implanted device.

	Overall (n=64)	***#E*** (n=24)	***#P*** (n=20)	***#wP*** (n=20)	***P value***
Baseline characteristics					
Age, years	80.9±5.3	80.3±5.3	82.0±5.6	80.5±6.4	0.561
Female sex	41 (64.1%)	15 (62.5%)	14 (70.0%)	12 (60.0%)	0.789
Arterial hypertension	51 (79.7%)	19 (79.2%)	17 (85.0%)	15 (75.0%)	0.732
Diabetes mellitus	17 (26. %)	6 (25.0%)	5 (25.0%)	6 (30.0%)	0.916
Atrial fibrillation	17 (27.4%)	7 (31.8%)	7 (35.0%)	3 (15.0%)	0.354
eGFR, mL/min	68.3±22.2	70.4±21.2	63.1±21.4	71.1±24.5	0.456
Prior aortic valvuloplasty	2 (3.1%)	0 (0.0%)	2 (10.0%)	0 (0.0%)	0.103
Severe chronic kidney disease*	6 (9.4%)	2 (8.3%)	3 (15.0%)	1 (5.0%)	0.542
Chronic obstructive pulmonary disease	18 (28.1%)	6 (25.0%)	4 (20.0%)	8 (40.0%)	0.339
Left ventricular ejection fraction, %	51.5±6.8	51.2±7.6	51.8±7.1	51.8±5.8	0.948
Mean aortic gradient, mmHg	52.4±17.3	47.2±11.4	51.6±15.2	59.5±22.7	0.060
Peak velocity, cm/m^2	4.6±0.7	4.4±0.6	4.5±0.4	5.0±0.9	0.283
Baseline therapy					
Aspirin	40 (67.8%)	15 (68.2%)	13 (65.0%)	12 (70.6%)	1.000
Clopidogrel	21 (35.6%)	8 (36.4%)	5 (25.0%)	8 (47.1%)	0.360
Warfarin	2 (3.4%)	2 (9.1%)	0	0	0.326
Novel oral anticoagulant	15 (25.4%)	5 (22.7%)	7 (35.0%)	3 (17.7%)	0.488
Dual antiplatelet therapy	18 (30.5%)	8 (36.4%)	4 (20.0%)	6 (35.3%)	0.464
Single antiplatelet agent plus anticoagulant	5 (8.5%)	2 (9.1%)	2 (10.0%)	1 (5.9%)	1.000
Triple antithrombotic therapy	2 (3.4%)	1 (4.6%)	1 (5.0%)	0	1.000
Procedural features					
Femoral access	63 (98.4%)	23 (95.8%)	20 (100.0%)	20 (100.0%)	0.429
Closure device use	60 (93.8%)	23 (95.8%)	20 (100.0%)	17 (85.0%)	0.127
Postdilation	37 (57.8%)	13 (54.2%)	11 (55.0%)	13 (65.0%)	0.734
Postprocedural paravalvular regurgitation**					0.873
None	32/42 (76.2%)	11/15 (73.3%)	12/16 (75.0%)	9/11 (81.8%)	
Mild	10/42 (23.8%)	4/15 (26.7%)	4/16 (25.0%)	2/11 (18.2%)	
Discharge features					
Aspirin	59 (93.7%)	22 (91.7%)	18 (94.7%)	19 (95.0%)	1.000

Clopidogrel	45 (71.4%)	16 (66.7%)	15 (79.0%)	14 (70.0%)	0.731
Warfarin	5 (7.9%)	4 (16.7%)	1 (5.3%)	0	0.146
Novel oral anticoagulant	16 (25.4%)	6 (25.0%)	6 (31.6%)	4 (20.0%)	0.668
Dual antiplatelet therapy	42 (66.7%)	14 (58.3%)	14 (73.7%)	14 (70.0%)	0.595
Single antiplatelet agent plus anticoagulant	21 (33.3%)	10 (41.7%)	7 (36.8%)	4 (20.0%)	0.316
Triple antithrombotic therapy	4 (6.3%)	1 (4.2%)	3 (15.0%)	0	0.190
Paravalvular regurgitation					0.802
None	38/63 (60.3%)	16/24 (66.7%)	11/19 (57.9%)	11/20 (55.0%)	0.709
Mild	23/63 (36.5%)	8/24 (33.3%)	7/19 (36.8%)	8/20 (40.0%)	0.900
Moderate	2/63 (3.2%)	0/24 (0.0%)	1/19 (5.3%)	1/20 (5.0%)	0.273

Values are expressed as mean±SD or n (%).
#P= Portico recipients; #P= Portico with extra-rinsing recipients; #E= Evolut recipients.
eGFR= Estimated glomerular filtration rate; **data available for 42 patients; §data available for 63 patients.

Table S3. Comparative overview of selected transcatheter aortic valve systems.

	EVOLUT PRO ***Medtronic***	***EVOLUT PRO+*** ***Medtronic***	***EVOLUT R*** ***Medtronic***	***PORTICO*** ***Abbott Structural Heart***
Delivery	Self-expanding	Self-expanding	Self-expanding	Self-expanding
Design	Supra-annular valve	Supra-annular valve	Supra-annular valve	Intra-annular valve
Frame	Nitinol	Nitinol	Nitinol	Nitinol
Leaflets	Porcine pericardium	Porcine pericardium	Porcine pericardium	Bovine pericardium
Sheath size	16 Fr	14 Fr (16 Fr for 34 mm prosthesis)	14 Fr (16 Fr for 34 mm prosthesis)	18/19 Fr (14/15 Fr with FlexNav system)
Prosthesis size	23-26-29	23-26-29-34 mm	23-26-29-34 mm	23-25-27-29 mm
Annular diameter	From 18 to 26 mm	From 18 to 30 mm	From 18 to 30 mm	From 19 to 27 mm
Storage solution	Glutaraldehyde	Glutaraldehyde	Glutaraldehyde	Formaldehyde
Recapturable	Yes	Yes	Yes	Yes
Repositionable	Yes	Yes	Yes	Yes
Retrievable	Yes	Yes	Yes	Yes
Comments	Compared with Evolut R, an outer pericardial skirtwasadded to reduceparavalvular leak	Evolut Pro+ iscompatible with smallersheaths and offers a34-mm valve with outerpericardial sheath		Large cell geometry and intra-annular leaflet position preserve coronary access, provides early valve function and hemodynamic stability throughout the procedure.
